# Influenza D Virus in Domestic and Stray Cats, Northern China, 2024

**DOI:** 10.3201/eid3108.250042

**Published:** 2025-08

**Authors:** Mingshuai Shen, Xinkun Zhao, Jian Zhang, Cun Liu, Chengyi Qi, Ruohan Wang, Jieshi Yu, Kezhou Wang, Zhao Wang

**Affiliations:** Shandong First Medical University, Jinan, China (M. Shen, X. Zhao, J. Zhang, C. Qi, R. Wang, K. Wang, Z. Wang); Shandong Provincial Center for Animal Disease, Jinan (C. Liu); Guangdong Academy of Agricultural Sciences, Guangzhou, China (J. Yu)

**Keywords:** Influenza D virus, viruses, influenza, respiratory infections, zoonoses, domestic cats, feral cats, feline, China

## Abstract

Influenza D virus infects primarily cattle, but infrequent reports of infections in cats occur. We detected influenza D virus antibodies in 8 of 360 cats in northern China. Domestic cats showed higher susceptibility than strays. Our results suggest a previously overlooked aspect of epidemiology of this virus in companion animals.

Influenza D virus (IDV) is a single-stranded, negative-sense RNA virus belonging to the genus *Deltainfluenzavirus*, family Orthomyxoviridae, and was first isolated from pigs in 2011 (*1*,*2*). IDV infects primarily cattle, but studies have documented the virus globally in a diverse range of animals, including small ruminants (e.g., sheep), domestic animals (pigs, horses, camels), and wild ungulates (wild boars) (*3*,*4*). Although there is no direct evidence that IDV can infect humans, an increasing number of studies have indicated that IDV has the potential for causing zoonotic infections (*5*). One recent study in the Puglia region of Italy identified 14 positive IDV antibody samples from 426 dogs sampled in 2016–2023, indicating that companion animals can be exposed to IDV (*6*). Our study aimed to investigate whether cats, a popular companion animal, are susceptible to IDV.

We collected 360 serum samples from cats in northern China in 2024, comprising 181 samples from routine health checks at pet hospitals and 179 samples from pet rescue stations. We stored all samples at −80°C for subsequent analysis. Prior to testing them, we treated all samples with receptor-destroying enzyme to remove nonspecific inhibitors (*7*). We briefly mixed the samples with receptor-destroying enzyme in a 1:3 ratio, incubated them at 37°C overnight (18–20 h), then heat-inactivated them at 56°C for 30–60 minutes before hemagglutination inhibition (HI) testing. We performed screening by HI assay to identify antibodies against influenza A virus (A/China/SWL1304/2023 [H1N1]), influenza B virus (B/Guangdong/266/2021), and IDV (D/bovine/CHN/JY3002/2022), considering HI titers ≥10 to be positive (*8*). To confirm specificity, we further subjected HI-positive samples to virus neutralization assays using 100 TCID_50_ (50% tissue culture infectious dose) of IDV propagated in Madin-Darby canine kidney cells. We deemed neutralization titers ≥10 (highest serum dilution showing ≥50% cytopathic effect reduction) to be confirmatory (*9*).

Serological analysis revealed 6 (3.31%) of 181 of veterinary hospital samples to be HI positive for IDV antibodies, with 4 (66.7%) confirmed by virus neutralization assay (titers 10–20). Among rescue station samples, 2 (1.12%) of 179 were HI positive, with 2 (100%) confirmed by virus neutralization (titer 10) (odds ratio 4.12, 95% CI 0.92–18.40; p = 0.027). All positive samples showed HI titers of 10–40, and none were reactive to influenza A virus or influenza B virus (Table).

**Table:** Hemagglutination inhibition and virus neutralization antibody titers against influenza D virus in feline serum samples from study of influenza D virus in domestic and stray cats, northern China, 2024*

Sample no.	Influenza A virus, A/China/SWL1304/2023(H1N1)		Influenza B virus, B/Guangdong/266/2021		Influenza D virus, D/bovine/CHN/JY3002/2022	
	HI		HI		HI	VN
A5	<10		<10		10, 10	10, 10
D5	<10		<10		20, 20	20, 20
D8	<10		<10		20, 20	10, 10
E3	<10		<10		10,10	<10
H1	<10		<10		40, 40	20, 20
I3	<10		<10		20, 20	10, 10
I6	<10		<10		10, 10	<10
L7	<10		<10		40, 40	20, 20

*Samples were tested against 3 strains: influenza A virus, influenza B virus, and influenza D virus. Titers below the detectable threshold (10) were indicated as <10 and considered negative. HI, hemagglutination inhibition; VN, virus neutralization.

Our findings demonstrated that, although overall IDV seroprevalence in cats in northern China was low (2.22%), domestic cats showed significantly higher exposure rates than strays. This difference might reflect increased human contact, potentially leading to viral exposure, considering stray cats’ independent lifestyles potentially limit such transmission opportunities. As noted in similar studies of other species, the source of IDV infection in cats remains unclear. Potential transmission routes might include reverse zoonotic transmission or alternative pathways, such as raw milk exposure, as suggested by recent influenza A(H5N1) virus detections in cow’s milk (*10*). Although our serologic data cannot confirm active transmission or clinical impacts, the observed higher seroprevalence in domestic cats suggests that close human–animal interaction could potentially increase exposure risk.

IDV is not currently a major zoonotic threat, but its detection in cats highlights the need for vigilance. Domestic cats may potentially serve as reservoirs for IDV, which could contribute to viral adaptation. Our study emphasizes the importance of monitoring IDV in companion animals, especially considering recent findings demonstrating the potential of influenza viruses for cross-species transmission via unconventional routes (i.e., H5N1 virus in bovine milk).

Future research should prioritize molecular confirmation of active infections and assess milkborne transmission risks. Proactive surveillance in pets and their food sources is critical to understanding IDV’s evolving epidemiology and mitigating potential public health concerns.
